# Source evaluation of ^137^Cs in foodstuffs based on trace ^134^Cs radioactivity measurements following the Fukushima nuclear accident

**DOI:** 10.1038/s41598-018-35183-z

**Published:** 2018-11-14

**Authors:** Mayumi Hori, Takuya Saito, Katsumi Shozugawa

**Affiliations:** 1 Komaba Organization for Educational Excellence, The University of Tokyo, 3-8-1 Komaba, Meguro, Tokyo, 153-8902 Japan; 2 Akita Radiation Measuring Station (Beguredenega), Katagami, Akita, 018-1400 Japan; 3 Graduate School of Arts and Sciences, The University of Tokyo, 3-8-1 Komaba, Meguro, Tokyo, 153-8902 Japan

## Abstract

We performed gamma-ray analysis to determine the amount of radioactive cesium-134 (^134^Cs) and cesium-137 (^137^Cs) in 259 foodstuffs five years after the Fukushima nuclear accident of 2011. Using measurements of trace ^134^Cs radioactivity, we investigated the contribution ratio of ^137^Cs derived from the Fukushima accident on 2011 and pre-Fukushima. The median detected concentration of radiocesium (^134^Cs + ^137^Cs) in foodstuffs was 0.33 Bq/kg-raw, a much lower radioactivity than the Japanese regulatory limit. However, a few samples had particularly high radioactivity, including some dried mushrooms sold in Iwate Prefecture that had a ^137^Cs radioactivity concentration as high as 441 Bq/kg. Our analysis showed that 75.5% of the ^137^Cs detected in these mushrooms originated from the Fukushima accident, and 24.5% was originated before the Fukushima event. Our study clarified the ^137^Cs contamination in 75 of all 259 food samples before and after the Fukushima nuclear accident, showing that not only mushrooms but also fish had been contaminated before the Fukushima accident.

## Introduction

The March 2011 accident at the Fukushima Daiichi nuclear power plant, TEPCO, in Japan caused the large release of several radionuclides, including cesium-134 and cesium-137, into the atmosphere and Pacific Ocean^1–4^, resulting in the contamination of various food products^5–8^. Five years after the Fukushima accident, the probability of foodstuffs containing radionuclide levels exceeding the Japanese regulatory radiocesium limit (general foods: 100 Bq/kg, valid since April 1, 2012^9,10^) was extremely low because of collaboration among farmers, producers, and agricultural organizations to ensure food safety. The survey conducted by Fukushima Prefecture from April 2015 to April 2016 revealed that 23,837 of the 23,855 foods (over 99.9%) produced in Fukushima had radiocesium concentrations below the regulatory limit^11^.

Although gamma-ray measurements supported that radioactivity levels in almost all foodstuffs were far below the Japanese regulatory limit, it was unknown how much of the detected radioactivity originated from the Fukushima accident as opposed to pre-Fukushima events such as the atmospheric nuclear explosions^12^ that have been conducted since 1945 and the Chernobyl nuclear power plant accident of 1986.

Before the Fukushima accident, gamma-ray measurements to determine anthropogenic radionuclides in Japanese foodstuffs were very limited^13–15^. Following the accident, measurements of food radioactivity levels, especially of ^137^Cs, became more readily available because of the Japanese government’s rapid establishment of a food monitoring campaign to detect radionuclides. While this campaign produced a large dataset of radiocesium contamination levels in food, no quantitative method existed to distinguish between the detected ^137^Cs that originated from the Fukushima accident and the detected ^137^Cs that originated from prior deliberate or accidental releases of the radionuclide.

In this study, we first performed gamma-ray analysis to investigate the distribution of radiocesium in 259 general foodstuffs five years after the Fukushima accident. Using the trace radioactivity of short-lived ^134^Cs in foodstuffs, we then evaluated the contribution ratio of Fukushima-derived ^137^Cs in general foodstuffs in Japan. Although ^134^Cs and ^137^Cs involve different generation processes in nuclear reactors and the ^134^Cs/^137^Cs activity ratio depends on the extent of fuel burnup in each reactor, their yield will be higher compared to other fission or activation products. Therefore, our method is applicable not only to the Fukushima accident but also to any future nuclear disaster.

## Results

**Figure 1:**
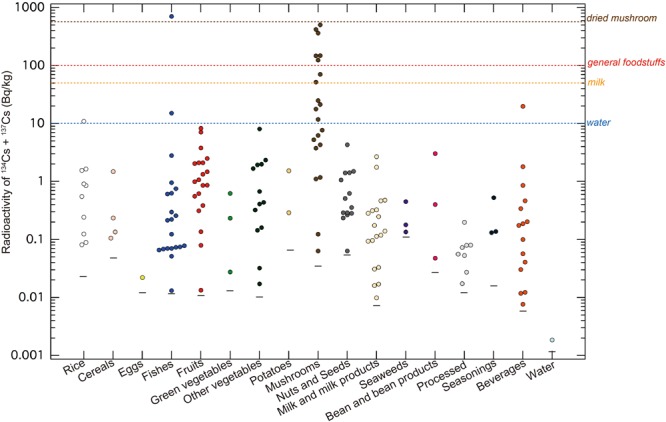
The detected radioactivity of the sum ^134^Cs and ^137^Cs in foodstuffs categorized into 17 groups, as measured in 2016, five years after the Fukushima accident. Each dashed colored line shows the Japanese regulatory limits for various foodstuff groups: water (10 Bq/kg), milk (50 Bq/kg), general foodstuffs (100 Bq/kg), and dried mushrooms (570 Bq/kg). The bars under the colored dots show the detection limits of each category. Radiocesium was detected in 161 samples, equivalent to 62.2% of all samples (n = 259).

Radioactivity of ^134^Cs and ^137^Cs in all 259 foodstuffs were analyzed during 2015–2016 period. For convenience in discussion, we considered this period as in 2016, five years after the Fukushima accident. Figure 1 shows the radioactivity of the sum of ^134^Cs and ^137^Cs in foodstuffs categorized into 17 groups (rice, cereals, eggs, fish, fruits, green vegetables, other vegetables, potatoes, mushrooms, nuts and seeds, milk and milk products, seaweeds, bean and bean products, processed foodstuffs, seasonings, beverages and water) according to our previous reports^6^. Most of these foodstuffs had total radioactivity levels (^134^Cs + ^137^Cs) below the Japanese regulatory limit for water of 10 Bq/kg, milk of 50 Bq/kg, general foodstuffs of 100 Bq/kg^9^, and dried Shiitake mushrooms (*Lentinula edodes*) of 570 Bq/kg^16^. Our analysis allowed the detection of much lower concentrations of radiocesium as compared to those of the screening level.

Of the all 259 foodstuff samples, 161 samples (62.2%) contained either both ^134^Cs and ^137^Cs or only ^137^Cs in concentrations above the detection limit. The sum of the radioactivity of ^134^Cs and ^137^Cs ranged from 1.86 × 10^−3^ to 707 Bq/kg. Among the cesium-detected sample set, the average sum of the radioactivity of ^134^Cs and ^137^Cs was 17.4 Bq/kg, and the median was 0.33 Bq/kg. These results indicate that very few samples had high radioactivity levels. The lowest radioactivity of the sum of ^134^Cs and ^137^Cs was found in tap water collected at Nerima City, Tokyo: 1.86 × 10^−3^ Bq/kg, which is equivalent to 1/5,380 of the Japanese regulatory limit. Conversely, a sample of shortfin mako shark (*Isurus oxyrinchus*) collected offshore from the Izu Peninsula was 707 Bq/kg, far exceeding the Japanese regulatory limit of 100 Bq/kg-raw. However, this case was very rare, as the maximum radioactivity in 559 fish samples collected offshore from the North Pacific Ocean in this period was 44 Bq/kg-raw^17^.

In the rice category, rice bran had the highest radioactivity (10.9 Bq/kg), although that of polished rice samples ranged from 0.08 to 0.91 Bq/kg. The radioactivity ratio of bran to polished rice was about 0.1, which was good agreement with the cultivation in Fukushima^18^.

Mushrooms tended to have higher radioactivity than other foods. The median radioactivity was only 1.2 Bq/kg-raw; however, the dried mushroom sample purchased at Iwate Prefecture in northern Fukushima showed the highest radioactivity, with 505 Bq/kg of radiocesium. Even the dried mushroom sample purchased at Kanagawa Prefecture, which is located 306 km from the Fukushima Daiichi Nuclear Power Plant, had a radioactivity concentration of 147 Bq/kg. In contrast, two samples of dried mushrooms purchased at Minami-Soma in Fukushima Prefecture had lower radioactivity (11.8 and 71.8 Bq/kg, respectively) than that of other prefectures. These measurements indicate that the source of contamination is not unique. Mushrooms are well-known as cesium accumulators^19–21^; thus, a continuous food monitoring campaign over long distances is required to ensure food safety.

**Figure 2:**
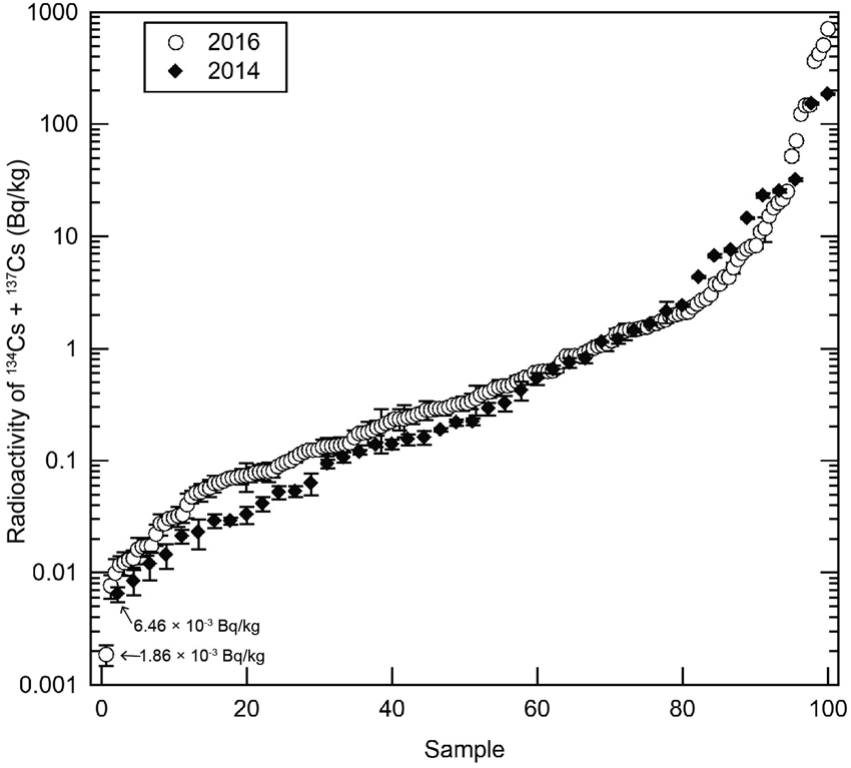
Comparison of distribution of the radioactivity of the detected sum of ^134^Cs and ^137^Cs (radiocesium) in 2014 (closed diamond) and 2016 (open circle), respectively, three and five years after the Fukushima accident. The number of samples was normalized to 100% on the horizontal axis for ease of comparison.

Figure 2 represents the radioactivity distribution of detected radiocesium five years after the Fukushima accident in 2016 (n = 161, ○), as compared with our previous data (n = 96, ◆) from 2014^6^, three years after the accident. The difference in analysis accuracy, precision, and contents of dataset between both the analyses was minimal. The number of samples was normalized to 100% on the horizontal axis for ease of comparison. The median radioactivity of radiocesium was 0.33 Bq/kg-raw in 2016 and 0.16 Bq/kg-raw in 2014. Although it seems that the distribution did not change much after two years, the comparison of median radioactivity is not statistically significant for two reasons: the dataset did not include the samples whose radioactivity concentrations were under the detection limit, and it was impossible to distinguish between the pre-Fukushima (before 2011) and post-Fukushima background.

## Discussion

Radiocesium, including ^134^Cs, could originate from pre-Fukushima events, such as atmospheric nuclear explosion fallout (since 1945), the Chernobyl nuclear power plant accident (1986), in addition to the Fukushima accident itself (2011). ^134^Cs originating from pre-Fukushima events is difficult to detect because 30 years have passed since the last event (Chernobyl accident), and its half-life (T_1/2_ = 2.06 years) is much shorter than that of ^137^Cs (T_1/2_ = 30.2 years).

In order to quantitatively estimate the ^134^Cs derived from the Chernobyl accident, the radioactivity ratio of ^134^Cs/^137^Cs at the time of the Chernobyl accident was used. The estimated ^134^Cs as of 1 January 2016 was 4.99 × 10^−5^, which was corrected according to the ^134^Cs/^137^Cs radioactivity ratio of 0.55 at the time of the Chernobyl accident^22,23^. This estimation indicated that ^134^Cs originating from the Chernobyl accident (pre-Fukushima) had decayed away nearly entirely by 2016, and thus it would be hardly detectable with any high-sensitivity HPGe detector.

In the case of chai leaves from Turkey that were sold in Japan, for example, the radioactivity of ^137^Cs was 19.9 Bq/kg, while that of ^134^Cs was below the detection limit (<0.195 Bq/kg). According to the ^134^Cs/^137^Cs ratio at the time of the Chernobyl accident, the ideal ^134^Cs radioactivity would be 1.76 mBq/kg at the time of our gamma-ray measurement. This analysis indicated that pre-Fukushima-derived ^134^Cs radioactivity was negligible after the Fukushima accident.

Therefore, in this study, all detected ^134^Cs in foodstuffs in Japan could be regarded as originating from the Fukushima accident, although detected ^137^Cs may be the result of pre-Fukushima events.

As previously discussed and previous study^24^, it is difficult to uniquely evaluate the impact of the Fukushima accident. The data in Figs 1 and 2 represent the combined influence of the Fukushima accident and pre-Fukushima events.

Thus, a quantitative model for ^137^Cs derived from Fukushima was developed to estimate the effect of the accident itself, based on the detected radioactivity of ^134^Cs in our study and the radioactivity ratio of ^134^Cs/^137^Cs at the time of the Fukushima accident. Although the ratios are slightly different for each reactor unit (1,2, and 3) in Fukushima nuclear power plant, the total ^134^Cs/^137^Cs ratio at the time of the accident could be regarded as roughly 1.0^25^. In addition, because we could regard all detected ^134^Cs in foodstuffs as originating from the Fukushima accident, the ratio simply varied according to the function of elapsed time, *t* (years), after stopping the generation of activation and fission products such as ^134^Cs and ^137^Cs. Therefore, ^137^Cs originating from the Fukushima accident, ^137^Cs_*Fukushima*_, could be written as

1 $${}^{137}C{s}_{Fukushima}=\,\frac{{}^{134}C{s}_{detected}}{{f}_{t}}$$

2 $${f}_{t}={2}^{t(\frac{1}{30.2}-\frac{1}{2.06})}$$

3 $${}^{137}C{s}_{PreFukushima}={}^{137}C{s}_{detected}-{}^{137}C{s}_{Fukushima}$$

where ^137^Cs_*Fukushima*_ is the radioactivity of ^137^Cs originating from the Fukushima accident, ^137^Cs_*PreFukushima*_ is the ^137^Cs originating from pre-Fukushima events such as the Chernobyl accident or global fallout, ^134^Cs_*detected*_ and ^137^Cs_*detected*_ are the radioactivity of ^134^Cs and ^137^Cs in food samples as detected by gamma-ray measurements, and *f*_*t*_ is the physical-decay function based on the specific ^134^Cs/^137^Cs radioactivity ratio of the Fukushima accident. The values of 30.2 and 2.06 indicate the T_1/2_ of ^137^Cs and ^134^Cs, respectively, and *t* is the elapsed time (in years) since the Fukushima accident on March 11, 2011. In this study, all ^137^Cs_*Fukushima*_ measurements were calculated based on decay-corrected radioactivity.

We could not determine that ^137^Cs_*detected*_ corresponded to ^137^Cs_*PreFukushima*_ in samples where ^134^Cs was not detected, as it is possible that the gamma-ray detector lacked the capabilities needed to detect the radioactivity of ^134^Cs. However, in samples where ^134^Cs was detected, we could clearly and quantitatively show whether Fukushima contributed to the radioactivity.

**Figure 3:**
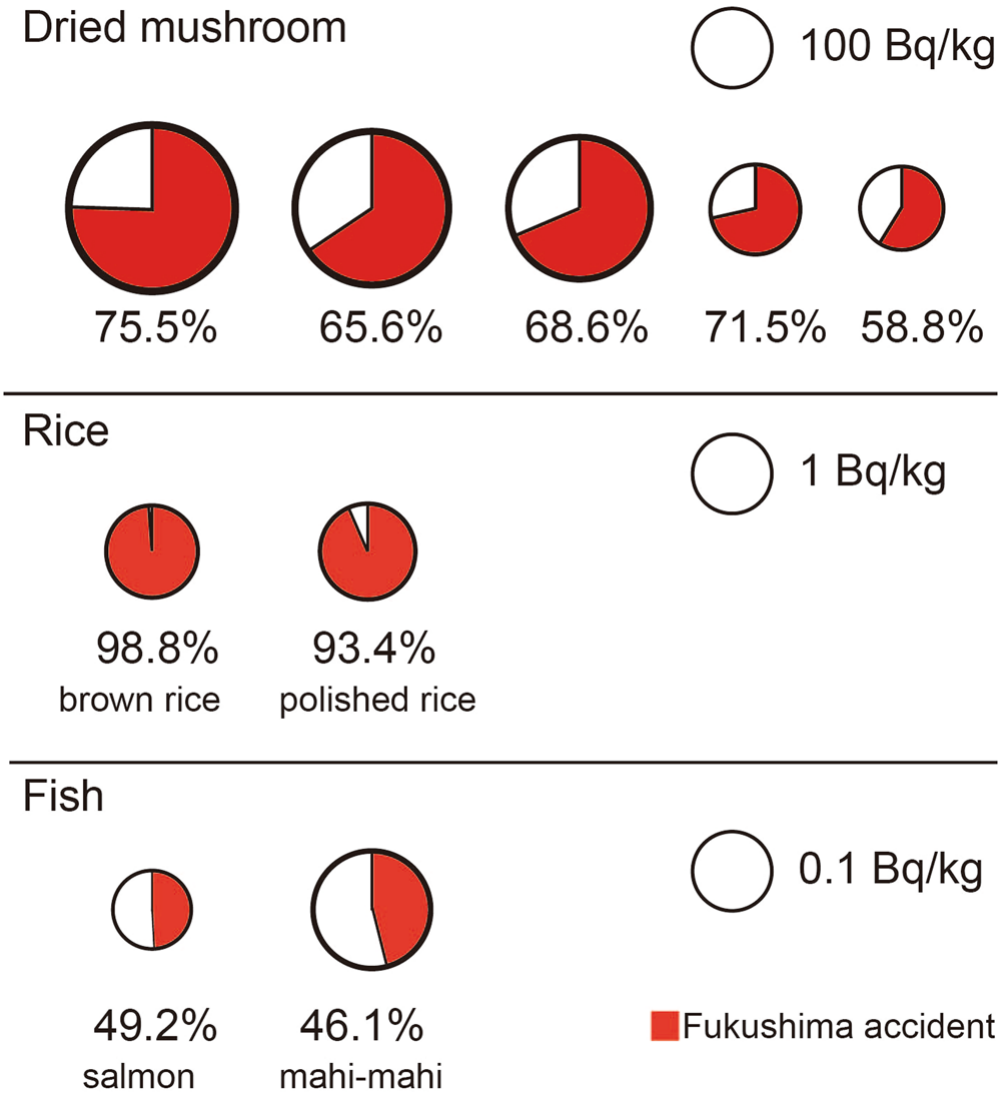
The specific and representative contribution ratio of ^137^Cs_*Fukushima*_ in Japanese foodstuffs. The area of the circles is proportional to the radioactivity of the corresponding foodstuffs. (See Supplementary Information: Table S1 in detail).

The representative contribution ratio of ^137^Cs_*Fukushima*_ after the Fukushima accident is shown in Fig. 3. The entire list of all samples can be found as Supplementary Table S1. Contribution ratio included about 18% on average. Because 1) the ^134^Cs/^137^Cs ratio at the time of accident was distributed from 0.9 to 1.1^25^, and 2) the smaller the radioactivity, the larger the error of ^134^Cs/^137^Cs ratio. Considering the two factors, the error of contribution ratio (1sigma) was 17.9 ± 6.8% in our dataset.

Samples in which the detected ^137^Cs originated from the Fukushima nuclear power plant were confirmed in 17 categories, regardless of the radioactivity concentration and research period.

Among the five samples of dried Shiitake mushrooms sold at the roadside rest area (Michi-no-eki) in Iwate Prefecture, ^137^Cs_*detected*_ ranged from 111 to 441 Bq/kg, and the percentage of ^137^Cs_*Fukushima*_ ranged from 58.8% to 75.5%. The mushroom with the highest radioactivity had 441 Bq/kg (^137^Cs) and 63.6 Bq/kg (^134^Cs). This mushroom was purchased on June 21, 2016, and thus, *f*_*t*_ was 0.191 because 5.28 years had passed since the Fukushima accident according to equation (2). As a result, 333 Bq/kg of ^137^Cs originated from Fukushima (^137^Cs_*Fukushima*_), and the other 108 Bq/kg of ^137^Cs was from a pre-Fukushima event (^137^Cs_*PreFukushima*_). Equivalently, 75.5% of ^137^Cs originated from the Fukushima accident. (If ^134^Cs was also included in the contribution, the percentage of Fukushima accident would be 78.5%).

For brown rice collected at Chiba Prefecture in 2016, for instance, the radioactivity of ^137^Cs_*Fukushima*_ was estimated to be 1.38 Bq/kg-raw, and ^137^Cs_*PreFukushima*_ was 0.017 Bq/kg-raw. In polished rice collected at the town of Namie in Fukushima Prefecture in 2014, ^137^Cs_*Fukushima*_ and ^137^Cs_*PreFukushima*_ were 1.75 Bq/kg-raw and 0.124 Bq/kg-raw, respectively. Thus, both samples had much lower radioactivity than the Japanese regulatory limit; however, the Fukushima accident strongly affected these radioactivity concentrations.

In contrast, fish had a relatively low concentration of ^137^Cs_*Fukushima*_, with the exception of the shortfin mako shark (*Isurus oxyrinchus*). In salmon collected at Hokkaido in 2014, ^137^Cs_*Fukushima*_ and ^137^Cs_*PreFukushima*_ were 50.9 mBq/kg-raw and 52.5 mBq/kg-raw, respectively. The contribution ratio of ^137^Cs from the Fukushima accident in these salmon was 49.2%. Fish, especially the migratory kind, might not be directly affected by the Fukushima accident. In the fish category of the 2016 dataset (Table S1), the contribution of ^137^Cs_*Fukushima*_ ranged from 46 to 100% (n = 10). The ratios of mahi-mahi (Dorado, *Coryphaena hippurus*) and tuna were 46% and 57%, respectively, indicating that the amount of ^134^Cs (and ^137^Cs) leaking into the Pacific Ocean from the Fukushima plant was limited five years after the accident.

In conclusion, we quantitatively evaluated the radioactive contamination caused by the Fukushima accident by conducting detailed gamma-ray analysis. Our results suggest that the ^137^Cs in mushrooms will continue to be detected for a long time. Because cesium accumulators such as mushrooms may be affected by pre-Fukushima events, it is necessary to perform gamma-ray measurements not only around Fukushima but also in other areas. In the area of eastern Japan especially, it would be important to estimate the contamination caused by the Fukushima accident and pre-Fukushima events.

Radioactive contamination of food in Japan as a result of the Fukushima accident is neither uniform across different food categories nor a function of distance from the Fukushima plant. Such contamination is not only due to the Fukushima accident but also to other previous events that released radiocesium. Therefore, we strongly insist that a continuous monitoring system be put in place to ensure Japanese food safety.

## Methods

### Food samples

All 259 foodstuff samples (except tap water) were purchased in 2016 at several supermarkets in Japan and through online shops. The country of origin of most samples is Japan, but countries of origin such as Russia, China and Turkey are also included as imported foods for comparison. Samples were categorized into 17 groups according to our previous method^6^ and some reports^26,27^: rice, cereals, eggs, fish, fruits, green vegetables, other vegetables, potatoes, mushrooms, nuts and seeds, milk and milk products, seaweeds, beans and bean products, processed foodstuffs, seasonings, beverages and water.

### Experimental design and procedure

To realize a lower detection limit, a concentration step based on the Japanese official method was added to the pre-treatment^28^. Samples with high water content, such as vegetables, were concentrated by freeze-drying and/or heat treatment around 80 degree for several days, as described in previous study^6^. In this study, the concentration (dry/wet) ratio ranged from 1.1 to 40. The dried samples were crushed by a mixer and packed into a U-8 plastic vessel (0.1 dm^3^) or Marinelli vessel (2.0 dm^3^) with a fixed geometry.

The radioactivity of each sample was determined using a high-purity germanium (HPGe) detector with a 25% relative efficiency at 1.33 MeV, and a resolution ranging from 1.80 keV to 1.33 MeV was used to collect the gamma rays emitted from the dried samples. A digital 16 k multichannel analyzer with an integrated high-voltage power supply was used for this system. The radioactivity of ^137^Cs was determined by the 661 keV gamma emission peak. For ^134^Cs, radioactivity were determined using the weighted average of the 604 keV and 795 keV gamma peaks after careful consideration of the sum effect. The measurements time (live time) depended mostly on each Compton scattering of ^40^K in each sample, and ranged from 2 hours to 13.2 days. Detection limits were set to three sigma (3σ) in accordance with Kaiser’s method^29^. Gamma rays measurement was continued until relative standard deviation reached 10% or less of its radioactivity. The detection limit also depended on the concentration of elements comprising the samples. In the case of water, the concentration (dry/wet) ratio was about 40, and the detection limit of ^137^Cs was 10^−3^ Bq/kg-raw. The radioactivity of ^134^Cs, ^137^Cs, and ^40^K were corrected (decay-backed) to the time of purchase of the foodstuffs.

To ensure quality control, we obtained radioactivity concentrations of the Japanese standard reference material JSAC 0471. The measurements—86.2 ± 1.8 Bq/kg (^134^Cs) and 112 ± 1.8 Bq/kg (^137^Cs)—were in good agreement with certified radioactivity concentrations (^134^Cs: 85.3 ± 5.9 Bq/kg and ^137^Cs: 115 ± 8 Bq/kg).

## Electronic supplementary material

Table S1
