# Supplementary material for: Source evaluation of 137Cs in foodstuffs based on trace 134Cs radioactivity measurements following the Fukushima nuclear accident
Source: Sci Rep. 2018 Nov 14;8:16806. doi: 10.1038/s41598-018-35183-z (PMC6235920; doi:10.1038/s41598-018-35183-z)
Supplement: Supplementary file 1 — Table S1 [file 41598_2018_35183_MOESM1_ESM.pdf]

Supplementary Information

Source evaluation of ^{137}Cs in foodstuffs based on trace ^{134}Cs radioactivity measurements following the Fukushima nuclear accident

Mayumi Hori, [1,*] Takuya Saito, [2] Katsumi Shozugawa[3, *]

[1] Komaba Organization for Educational Excellence, The University of Tokyo, 3-8-1 Komaba, Meguro, Tokyo 153-8902, Japan

[2] Akita Radiation Measuring Station (Beguredenega), Katakami, Akita 018-1400, Japan

[3] Graduate School of Arts and Sciences, The University of Tokyo, 3-8-1 Komaba, Meguro, Tokyo 153-8902, Japan

*Corresponding authors
Mayumi Hori
Phone: +81-3-5465-8242
E-mail: cmayumi@mail.ecc.u-tokyo.ac.jp

Katsumi Shozugawa
Phone&Fax: +81-3-5454-6566
E-mail: cshozu@mail.ecc.u-tokyo.ac.jp

Table S1: Dataset of radioactivity in foodstuffs after the Fukushima nuclear accident. Red letters in Detected ^{134}Cs column show N.D.(not detected). "-" in Ratio of Fukushima column show N.A. because of negligible small radioactivity of ^{134}Cs in each sample.

Year	category	Sample	Product information/origin	Distance from FDNPP (km)	decay back (yyyy/mm/dd)	Radioactivity sum of ^{134}Cs and ^{137}Cs (Bq/kg-raw)	error of radioactivity sum of ^{134}Cs and ^{137}Cs	Detected ^{134}Cs (Bq/kg-raw)	error of ^{134}Cs	Detected ^{137}Cs (Bq/kg-raw)	error of ^{137}Cs	$^{137}Cs_{Fukushima}$ (Bq/kg-raw)	Ratio of Fukushima (%)
	Bean and Bean products	Soybean	from Yamagata	99	2015/4/24	3.05.E+00	6.47.E-02	5.48.E-01	2.69.E-02	2.50.E+00	5.88.E-02	1.99.E+00	79.7
		dried natto	mixed from Ibaraki, Tochigi	-	2015/6/22	4.03.E-01	4.62.E-02	1.46.E-01		4.03.E-01	4.62.E-02		-
		Miso (Soybean paste)	(unknown)	-	2016/9/15	4.75.E-02	6.26.E-03	2.74.E-02		4.75.E-02	6.26.E-03		-
	Beverages	Juice (peach)	by FUJIYA CO., LTD.	-	2016/2/4	1.75.E-01	5.38.E-03	3.57.E-02	2.28.E-03	1.39.E-01	4.87.E-03	1.66.E-01	100
		Juice (tomato)	from Fukushima, by KAGOME CO.,LTD.	-	2015/5/15	1.87.E-01	4.84.E-03	4.10.E-02	2.39.E-03	1.46.E-01	4.21.E-03	1.52.E-01	100
		Juice (peach)	from Fukushima	-	2015/11/27	8.62.E-01	2.16.E-02	1.69.E-01	7.95.E-03	6.93.E-01	2.01.E-02	7.41.E-01	100
		Juice (apple)	from Gumma	189	2015/8/5	1.81.E+00	1.24.E-01	3.83.E-01	5.00.E-02	1.43.E+00	1.14.E-01	1.51.E+00	100
		Beer	(unknown)	-	2016/7/30	7.70.E-03	1.83.E-03	6.00.E-03		7.70.E-03	1.83.E-03		-
		Juice (apple)	from Nagano	260	2015/8/7	1.18.E-02	2.25.E-03	8.40.E-03		1.18.E-02	2.25.E-03		-
		Juice (vegetable)	by Kirin Company, Limited	-	2016/7/25	1.23.E-02	2.92.E-03	9.68.E-03		1.23.E-02	2.92.E-03		-
		Juice	by Yakult Honsha Co.,Ltd.	-	2016/5/27	3.04.E-02	2.78.E-03	1.38.E-02		3.04.E-02	2.78.E-03		-
		Juice	by Calpis Co., Ltd.	-	2015/9/9	4.07.E-02	1.01.E-02	3.10.E-02		4.07.E-02	1.01.E-02		-
		Juice	by Suntory Holdings Limited	-	2015/9/18	5.72.E-02	1.03.E-02	3.61.E-02		5.72.E-02	1.03.E-02		-
		Green tea	by ITO EN, LTD.	-	2015/6/3	1.00.E-01	1.58.E-03	6.73.E-03		1.00.E-01	1.58.E-03		-
		Genmaicha (tea with roastedrice)	from Yamaguchi	935	2016/7/3	2.03.E-01	8.62.E-02	1.43.E-01		2.03.E-01	3.72.E-02		-
		Tea (assam tea)	from India	6213	2016/3/31	3.42.E-01	5.32.E-02	1.96.E-01		3.42.E-01	5.32.E-02		-
		Tea (rose hips)	from Republic of Chile	17132	2015/9/24	4.66.E-01	4.95.E-02	1.49.E-01		4.66.E-01	4.95.E-02		-
		Chai	from Turkey	8643	2015/11/9	1.99.E+01	2.16.E-01	1.96.E-01		1.99.E+01	2.16.E-01		-
	Cereals	Brown rice flakes	by Kellogg(Japan)G.K.	-	2016/6/6	1.48.E+00	3.61.E-02	2.44.E-01	1.31.E-02	1.23.E+00	3.36.E-02	1.26.E+00	100
		Flour	from Iwate	254	2016/8/4	1.06.E-01	1.12.E-02	2.66.E-02		1.06.E-01	1.12.E-02		-
		Wheat	(unknown)	-	2016/6/24	1.35.E-01	1.88.E-02	6.59.E-02		1.35.E-01	1.88.E-02		-
		Barley	from Ibaraki	131	2016/4/14	2.35.E-01	4.99.E-02	1.41.E-01		2.35.E-01	4.99.E-02		-
	Eggs	Eggs	from Machida, Tokyo	253	2015/3/30	2.22.E-02	4.77.E-03	1.82.E-02		2.22.E-02	4.77.E-03		-
	Fishes	Japanese pond smelt	from Ibaraki	160	2016/2/26	1.52.E+01	7.60.E-01	2.82.E+00	2.67.E-01	1.24.E+01	7.12.E-01	1.34.E+01	100
		Shortfin mako shark (*Isurus oxyrinchus*)	from Shizuoka, the Izu peninsula	707	2016/5/19	7.07.E+02	1.50.E+00	1.17.E+02	4.30.E-01	5.90.E+02	1.44.E+00	5.95.E+02	100
		Dried sardines	from Ibaraki, the Pacific Ocean	127	2016/2/12	9.58.E-01	3.80.E-02	1.55.E-01	1.74.E-02	8.03.E-01	3.37.E-02	7.26.E-01	90.5
		Greeneyes fish	from Ibaraki, the Pacific Ocean	70	2015/8/13	7.57.E-01	2.95.E-02	1.39.E-01	1.24.E-02	6.18.E-01	2.68.E-02	5.56.E-01	90.0
		Oister	from Miyagi, collected in 2015, then frozen	95	2016/6/7	7.04.E-02	2.58.E-03	1.03.E-02	1.27.E-03	6.01.E-02	2.24.E-03	5.34.E-02	88.9
		Bonito	from Miyagi, the Pacific Ocean	95	2015/4/28	6.10.E-01	1.07.E-02	1.02.E-01	4.50.E-03	5.07.E-01	9.66.E-03	3.73.E-01	73.4
		Tuna	from Shizuoka, the Pacific Ocean	374	2016/3/24	6.33.E-01	2.45.E-02	6.65.E-02	1.66.E-02	5.67.E-01	1.80.E-02	3.23.E-01	56.9
		Squid	from Aomori	350	2016/2/17	6.89.E-02	2.67.E-03	7.43.E-03	1.42.E-03	6.14.E-02	2.26.E-03	3.49.E-02	56.8
		Dorado	from Chiba (except bones/the internal organs)	217	2015/6/1	2.99.E-01	1.53.E-02	3.26.E-02	9.15.E-03	2.66.E-01	1.23.E-02	1.23.E-01	46.1
		Mackerel	from Iwate	120	2015/7/2	1.24.E-01	5.42.E-03	1.32.E-02	2.66.E-03	1.11.E-01	4.72.E-03	5.09.E-02	45.9
		Pacific saury	from Iwate, collected in 2014, then frozen	300	2015/11/13	1.31.E-02	1.23.E-03	1.71.E-02		1.31.E-02	1.23.E-03		-

		Flatfish	from Miyagi, Pacific Ocean	95	2015/8/1	5.17.E-02	5.91.E-03	1.96.E-02		5.17.E-02	5.91.E-03		-
		Mackerel	(unknown)	-	2015/4/25	6.59.E-02	5.32.E-03	1.91.E-02		6.59.E-02	5.32.E-03		-
		Whitebait	from Miyazaki	1071	2016/7/5	7.06.E-02	5.13.E-03	1.76.E-02		7.06.E-02	5.13.E-03		-
		Mackerel canned	(unknown)	-	2015/9/9	7.33.E-02	2.10.E-02	5.69.E-02		7.33.E-02	2.10.E-02		-
		Salmon	from Hokkaido	490	2016/3/16	7.81.E-02	9.37.E-03	2.28.E-02		7.81.E-02	9.37.E-03		-
		Tuna	from Okinawa	1772	2016/8/2	2.15.E-01	1.17.E-02	5.62.E-02		2.15.E-01	1.17.E-02		-
		Bonito	from Shizuoka	360	2016/5/13	2.22.E-01	7.11.E-03	2.06.E-02		2.22.E-01	7.11.E-03		-
		Tuna	from Indonesia	5500	2015/8/11	2.58.E-01	1.22.E-02	3.23.E-02		2.58.E-01	1.22.E-02		-
		Bonito (Dried bonito shavings)	(unknown)	-	2016/6/15	2.80.E+00	4.41.E-02	1.59.E-01		2.80.E+00	4.41.E-02		-
		Blueberries	from Ibaraki	181	2015/9/4	1.36.E-01	6.93.E-03	2.92.E-02	3.59.E-03	1.07.E-01	5.93.E-03	1.19.E-01	100
		Persimmon	from Aizuwakamatsu, Fukushima	98	2016/2/1	3.86.E-01	1.78.E-02	7.20.E-02	7.47.E-03	3.14.E-01	1.61.E-02	3.19.E-01	100
		Pear	from Chiba	205	2015/10/12	6.20.E-01	4.65.E-02	1.35.E-01	2.68.E-02	4.85.E-01	3.80.E-02	5.70.E-01	100
		Apple (peel)	from Gumma	189	2016/2/23	8.60.E-01	6.72.E-02	1.72.E-01	3.56.E-02	6.88.E-01	5.70.E-02	8.12.E-01	100
		Apple (core)	from Gumma	189	2016/2/23	1.00.E+00	5.82.E-02	1.94.E-01	3.10.E-02	8.09.E-01	4.93.E-02	9.15.E-01	100
		Blueberries	from Gumma	189	2016/8/5	1.47.E+00	1.13.E-01	2.87.E-01	6.71.E-02	1.18.E+00	9.06.E-02	1.56.E+00	100
		Apple (peel)	from Fukushima-city, Fukushima	62	2015/12/5	2.10.E+00	6.95.E-02	4.02.E-01	3.04.E-02	1.70.E+00	6.25.E-02	1.77.E+00	100
		Blueberries	from Gumma	189	2015/7/27	8.64.E-01	1.03.E-01	2.36.E-01	4.95.E-02	6.28.E-01	9.03.E-02	9.32.E-01	100
		Rasberry	from Setagaya, Tokyo	231	2015/5/20	2.50.E+00	8.45.E-02	5.41.E-01	3.35.E-02	1.96.E+00	7.75.E-02	2.01.E+00	100
		Dried persimmon	from Gumma	189	2016/2/23	8.28.E+00	5.90.E-01	1.63.E+00	2.53.E-01	6.65.E+00	5.34.E-01	7.69.E+00	100
	Fruits	Apple	from Gumma	189	2016/2/23	1.07.E+00	4.49.E-02	1.84.E-01	1.70.E-02	8.88.E-01	4.16.E-02	8.70.E-01	97.9
		Apple	from Fukushima-city, Fukushima	62	2015/12/3	2.04.E+00	5.56.E-02	3.67.E-01	2.01.E-02	1.67.E+00	5.18.E-02	1.62.E+00	96.9
		Persimmon	from Gumma	210	2016/8/10	3.13.E-01	9.04.E-03	4.68.E-02	4.84.E-03	2.67.E-01	7.64.E-03	2.56.E-01	95.9
		Grapes	from Nihonmastu, Fukushima	56	2015/10/16	2.11.E+00	1.23.E-01	3.84.E-01	5.80.E-02	1.72.E+00	1.08.E-01	1.62.E+00	94.2
		Rasberry	from Setagaya, Tokyo	231	2016/7/29	3.78.E+00	2.15.E-01	6.45.E-01	8.83.E-02	3.13.E+00	1.96.E-01	2.40.E+00	76.6
		Mango	from Thai land	4600	2015/9/28	1.34.E-02	2.24.E-03	9.65.E-03		1.34.E-02	2.24.E-03		-
		Figs	from Turkey	8643	2016/4/15	7.96.E-02	1.26.E-02	3.38.E-02		7.96.E-02	1.26.E-02		-
		Persimmon (peel)	from Aizuwakamatsu, Fukushima	98	2015/12/14	5.58.E-01	8.02.E-02	3.06.E-01		5.58.E-01	8.02.E-02		-
		Blueberries	from Hokota, Ibaraki	148	2015/9/3	1.34.E+00	1.57.E-01	5.26.E-01		1.34.E+00	1.57.E-01		-
		Blueberries	from Poland	8600	2015/10/16	7.09.E+00	2.49.E-01	2.36.E-01		7.09.E+00	2.49.E-01		-
	Green vegetables	Chainese cabbage	from Ibaraki	127	2016/5/6	2.75.E-02	2.52.E-03	5.44.E-03	2.12.E-03	2.21.E-02	1.36.E-03	2.74.E-02	100
		Chainese cabbage	from Hokota, Ibaraki	148	2016/1/27	2.34.E-01	2.53.E-02	9.91.E-02		2.34.E-01	2.53.E-02		-
		Japanese parsley (*Seri)*	from Yamanashi	263	2015/4/12	6.28.E-01	5.78.E-02	2.07.E-01		6.28.E-01	5.78.E-02		-
		Milk	from Iwate	225	2015/12/10	1.40.E-01	9.30.E-03	2.85.E-02	4.96.E-03	1.11.E-01	7.86.E-03	1.26.E-01	100
		Milk	from Iwate, by KOIWAI DAIRY PRODUCTS CO., LTD.	258	2015/4/11	4.66.E-01	4.35.E-03	9.88.E-02	1.61.E-03	3.67.E-01	4.04.E-03	3.55.E-01	96.8
		Milk	from Fukushima	60	2015/6/23	9.39.E-02	4.77.E-03	1.89.E-02	3.04.E-03	7.50.E-02	3.67.E-03	7.24.E-02	96.5
		Skimmed milk	by Morinaga company	-	2015/9/15	1.76.E+00	7.87.E-02	3.33.E-01	3.44.E-02	1.43.E+00	7.07.E-02	1.37.E+00	95.9
		Milk	by Meiji Co., Ltd.	-	2015/7/1	3.26.E-01	1.80.E-02	6.36.E-02	8.35.E-03	2.62.E-01	1.59.E-02	2.45.E-01	93.6
		Milk	from Fukushima	69	2015/8/13	3.16.E-01	2.02.E-02	5.95.E-02	7.61.E-03	2.56.E-01	1.87.E-02	2.38.E-01	92.9
		Milk	by MEGMILK SNOW BRAND Co.,Ltd.	-	2015/10/29	1.76.E-01	1.20.E-02	2.89.E-02	5.75.E-03	1.47.E-01	1.05.E-02	1.24.E-01	84.2
		Milk	from Tochigi	137	2015/8/15	1.19.E-01	4.71.E-03	2.03.E-02	2.13.E-03	9.86.E-02	4.20.E-03	8.14.E-02	82.6
		Milk	from Nemuro-Kushiro area, Hokkaido	750	2015/7/24	1.14.E-01	4.75.E-03	1.27.E-02	2.79.E-03	1.01.E-01	3.84.E-03	5.00.E-02	49.5
	Milk and Milk products	Powdered milk	(unknown)	-	2016/8/23	2.70.E+00	3.59.E-02	1.46.E-01	1.51.E-02	2.56.E+00	3.26.E-02	8.04.E-01	31.4
		Milk	from Shimane	766	2015/9/30	9.93.E-03	3.26.E-03	1.17.E-02		9.93.E-03	3.26.E-03		-
2016		Milk	from Fukui	455	2016/2/12	1.61.E-02	4.27.E-03	1.16.E-02		1.61.E-02	4.27.E-03		-
		Milk	from Tokachi, Hokkaido	652	2015/10/30	1.71.E-02	3.22.E-03	1.35.E-02		1.71.E-02	3.22.E-03		-

	Milk	from Yatsuren company	-	2015/12/31	3.08.E-02	3.50.E-03	1.34.E-02		3.08.E-02	3.50.E-03		-
	Yoghurt	by Morinaga company	-	2015/11/17	3.30.E-02	5.30.E-03	1.83.E-02		3.30.E-02	5.30.E-03		-
	Milk	from Hakodate, Hokkaido	485	2015/9/23	9.71.E-02	6.97.E-03	2.09.E-02		9.71.E-02	6.97.E-03		-
	Powdered milk	by Morinaga company	-	2015/11/25	2.49.E-01	3.85.E-02	1.53.E-01		2.49.E-01	3.85.E-02		-
	Powdered milk	by Morinaga company	-	2015/11/24	2.84.E-01	3.48.E-02	1.31.E-01		2.84.E-01	3.48.E-02		-
	Powdered milk	by Bean Stalk Snow Co.,Ltd.	-	2015/11/18	4.78.E-01	4.47.E-02	1.45.E-01		4.78.E-01	4.47.E-02		-
Mushrooms	Log-grown Shiitake mushrooms	from Ibaraki	152	2016/5/26	2.50.E+01	5.15.E-01	4.18.E+00	1.77.E-01	2.09.E+01	4.84.E-01	2.14.E+01	100
	Cortinarius praestans	from Miyagi	95	2016/1/26	1.79.E+01	5.05.E-01	3.21.E+00	1.59.E-01	1.47.E+01	4.79.E-01	1.48.E+01	100
	mushrooms	from Iwate	216	2016/8/15	5.19.E+01	3.90.E+00	8.47.E+00	1.77.E+00	4.34.E+01	3.47.E+00	4.65.E+01	100
	Dried Shiitake mushrooms	from Minami-soma, Fukushima	25	2015/6/11	7.10.E+01	1.63.E+00	1.54.E+01	6.61.E-01	5.56.E+01	1.49.E+00	5.83.E+01	100
	Shiitake mushrooms	from Gumma	189	2016/2/23	2.14.E+01	6.04.E-01	3.71.E+00	2.02.E-01	1.77.E+01	5.69.E-01	1.75.E+01	99.2
	Shiitake mushrooms	from Kanagawa, Shiitake mushroom	306	2016/1/4	1.47.E+02	6.06.E-01	2.63.E+01	1.85.E-01	1.20.E+02	5.77.E-01	1.19.E+02	98.8
	Shiitake mushrooms	from Gumma	189	2016/8/5	6.23.E+00	1.49.E-01	9.24.E-01	5.34.E-02	5.30.E+00	1.39.E-01	5.03.E+00	94.7
	Eryngii Mushrooms	from Niigata	166	2015/5/27	6.36.E-02	3.40.E-03	1.17.E-02	1.53.E-03	5.19.E-02	3.04.E-03	4.36.E-02	84.0
	Dried Shiitake mushrooms	from Iwate	239	2016/6/21	5.05.E+02	1.24.E+01	6.36.E+01	3.35.E+00	4.41.E+02	1.20.E+01	3.33.E+02	75.5
	Dried Shiitake mushrooms	from Iwate	239	2016/6/7	1.49.E+02	1.09.E+01	1.81.E+01	3.03.E+00	1.31.E+02	1.04.E+01	9.35.E+01	71.5
	Dried Shiitake mushrooms	from Iwate	239	2016/6/14	3.65.E+02	1.07.E+01	4.25.E+01	2.89.E+00	3.22.E+02	1.03.E+01	2.21.E+02	68.6
	Dried Shiitake mushrooms	from Iwate	239	2016/6/21	4.23.E+02	1.30.E+01	4.71.E+01	3.31.E+00	3.76.E+02	1.26.E+01	2.47.E+02	65.6
	Dried Shiitake mushrooms	from Iwate	239	2016/6/21	1.24.E+02	6.00.E+00	1.25.E+01	1.70.E+00	1.11.E+02	5.76.E+00	6.55.E+01	58.8
	Shiitake mushrooms	from Fukuoka	1025	2015/7/10	1.24.E-01	8.59.E-03	2.77.E-02		1.24.E-01	8.59.E-03		-
	Nameko mushrooms	from Gumma	189	2016/8/5	5.81.E-01	1.64.E-01	5.25.E-01		5.81.E-01	1.64.E-01		-
	Maitake mushrooms	from Niigata	189	2016/1/26	1.18.E+00	1.20.E-01	4.12.E-01		1.18.E+00	1.20.E-01		-
	Dried Maitake mushrooms (Hen of the Woods)	from Hokkaido	491	2015/6/27	3.77.E+00	3.84.E-01	1.35.E+00		3.77.E+00	3.84.E-01		-
	Porcini	from Spain	10653	2015/8/12	4.32.E+00	1.72.E-01	2.86.E-01		4.32.E+00	1.72.E-01		-
	Dried Shiitake mushrooms	(unknown)	-	2016/8/29	5.24.E+00	5.80.E-01	2.17.E+00		5.24.E+00	5.80.E-01		-
	Log-grown Shiitake mushrooms	mixed from Miyazaki, Oita and Ehime	-	2015/7/11	7.68.E+00	9.88.E-02	1.46.E-01		7.68.E+00	9.88.E-02		-
	Dried Shiitake mushrooms	from Minami-soma, Fukushima	25	2015/8/10	1.18.E+01	3.00.E+00	8.45.E+00		1.18.E+01	3.00.E+00		-
Nuts and Seeds	Chestnuts	from Ibaraki	156	2015/11/9	4.33.E+00	1.19.E-01	8.66.E-01	4.59.E-02	3.47.E+00	1.09.E-01	3.74.E+00	100
	Peanuts	from Chiba	217	2015/7/27	1.41.E+00	6.83.E-02	2.57.E-01	2.72.E-02	1.15.E+00	6.27.E-02	1.02.E+00	88.4
	Chestnuts	from China	3274	2015/11/6	6.31.E-02	1.09.E-02	3.33.E-02		6.31.E-02	1.09.E-02		-
	Coffee	(unknown)	-	2016/1/21	2.36.E-01	7.49.E-02	2.06.E-01		2.36.E-01	7.49.E-02		-
	Cocoa	(unknown)	-	2015/6/6	2.65.E-01	2.99.E-02	1.21.E-01		2.65.E-01	2.99.E-02		-
	Cocoa	by Morinaga company	-	2016/3/23	2.83.E-01	5.52.E-02	2.18.E-01		2.83.E-01	5.52.E-02		-
	Cocoa	by Morinaga company	-	2015/6/19	2.92.E-01	3.38.E-02	1.30.E-01		2.92.E-01	3.38.E-02		-
	Coffee	(unknown)	-	2015/2/27	2.92.E-01	3.72.E-02	1.12.E-01		2.92.E-01	3.72.E-02		-
	Coffee	(unknown)	-	2016/5/1	3.56.E-01	1.07.E-01	3.25.E-01		3.56.E-01	1.07.E-01		-
	Coffee	(unknown)	-	2015/3/19	5.10.E-01	5.94.E-02	2.17.E-01		5.10.E-01	5.94.E-02		-
	Coffee	Instant coffee produced before March 2011	-	2015/5/29	6.24.E-01	5.90.E-02	2.16.E-01		6.24.E-01	5.90.E-02		-
	Peanuts	from Chiba	217	2016/1/29	1.06.E+00	1.13.E-01	3.88.E-01		1.06.E+00	1.13.E-01		-
	Peanuts	from Chiba	217	2016/2/2	1.43.E+00	2.38.E-01	1.08.E+00		1.43.E+00	2.38.E-01		-
	Macadamia nuts	from Australia	7020	2016/2/9	1.50.E+00	2.92.E-02	8.00.E-02		1.50.E+00	2.92.E-02		-
	Edamame (Green soybeans)	from Gumma	189	2016/8/5	1.94.E+00	1.27.E-01	3.01.E-01	7.09.E-02	1.64.E+00	1.06.E-01	1.64.E+00	100
	Edamame (Green soybeans, peel)	from Gumma	189	2016/8/5	1.99.E+00	7.84.E-02	3.19.E-01	3.86.E-02	1.67.E+00	6.83.E-02	1.73.E+00	100
	Cucumber	from Hokota, Ibaraki	148	2015/9/8	4.38.E-01	2.74.E-02	9.63.E-02	1.58.E-03	3.41.E-01	2.74.E-02	3.92.E-01	100
	Myoga ginger	from Gumma	189	2016/8/5	8.12.E+00	3.14.E-01	1.27.E+00	1.07.E-01	6.85.E+00	2.95.E-01	6.90.E+00	100
	Sprouts of Aralia elata	from Gumma	189	2016/2/23	2.35.E+00	7.58.E-02	4.44.E-01	3.52.E-02	1.91.E+00	6.71.E-02	2.10.E+00	100

Other vegetables	Tea leaves	from Kakegawa, Shizuoka	401	2015/6/2	1.68.E+00	6.83.E-02	3.61.E-01	3.18.E-02	1.32.E+00	6.04.E-02	1.36.E+00	100
	Carrots	from Chiba	217	2015/7/21	1.44.E-01	6.19.E-03	2.76.E-02	3.38.E-03	1.16.E-01	5.18.E-03	1.08.E-01	93.3
	Japanese white radish	from Toyama	350	2015/3/25	1.72.E-02	3.08.E-03	9.71.E-03		1.72.E-02	3.08.E-03		-
	Lotus Root	from Yamaguchi	935	2015/3/26	3.22.E-02	6.42.E-03	2.42.E-02		3.22.E-02	6.42.E-03		-
	Japanese white radish	from Hokota, Ibaraki	148	2015/12/18	1.61.E-01	2.35.E-02	7.36.E-02		1.61.E-01	2.35.E-02		-
	Sprouts of Aralia elata	from Tainai, Niigata	160	2015/5/2	3.24.E-01	4.65.E-02	1.87.E-02		3.24.E-01	4.65.E-02		-
	Ostrich fern	from Tainai, Niigata	160	2015/5/2	4.10.E-01	4.55.E-02	1.61.E-01		4.10.E-01	4.55.E-02		-
	Green tea leaves	from Kagoshima	1160	2015/6/8	6.73.E-01	6.21.E-02	1.99.E-01		6.73.E-01	6.21.E-02		-
Potatoes	Sweet potato	from Ibaraki	127	2015/4/9	1.53.E+00	2.67.E-02	3.33.E-01	1.04.E-02	1.20.E+00	2.46.E-02	1.68.E+00	100
	Taro	from Yamagata	112	2016/3/14	2.90.E-01	9.61.E-03	4.81.E-02	4.22.E-03	2.42.E-01	8.64.E-03	2.31.E-01	95.4
Processed	Cake of ground fish combined with starch and steamed	by Kibun company	-	2016/4/22	5.61.E-02	2.82.E-03	7.77.E-03	1.74.E-03	4.83.E-02	2.22.E-03	3.86.E-02	79.9
	Boiled fish paste	by Kibun company	-	2016/7/7	7.26.E-03	2.48.E-03	1.02.E-02		7.26.E-03	2.48.E-03		-
	Minced fish	(unknown)	-	2016/8/3	2.74.E-02	5.91.E-03	2.43.E-02		2.74.E-02	5.91.E-03		-
	Snack	by KAMEDA SEIKA CO., LTD.	-	2016/6/16	7.45.E-02	9.95.E-03	3.95.E-02		7.45.E-02	9.95.E-03		-
	Chocolate snack	by BANDAI CO., LTD.	-	2016/8/19	5.28.E-02	9.75.E-03	5.32.E-02		5.28.E-02	9.75.E-03		-
	Wheat bread	by bakery in Tokyo	-	2016/4/11	7.30.E-02	1.17.E-02	4.74.E-02		7.30.E-02	1.17.E-02		-
	Biscuits	by FUJIYA CO., LTD.	-	2016/1/19	7.97.E-02	1.33.E-02	5.37.E-02		7.97.E-02	1.33.E-02		-
	Cat food	by Unicharm Corporation	-	2016/7/20	8.01.E-02	1.60.E-02	7.08.E-02		8.01.E-02	1.60.E-02		-
	Snack	by Calbee, Inc.	-	2016/7/24	1.98.E-01	3.05.E-02	1.12.E-01		1.98.E-01	3.05.E-02		-
Rice	Brown rice	from Chiba	217	2016/2/26	2.44.E-01	8.15.E-03	4.36.E-02	3.95.E-03	2.01.E-01	7.12.E-03	2.07.E-01	100
	Brown rice	from Tamura, Fukushima	40	2016/8/19	1.56.E+00	8.74.E-02	2.57.E-01	3.28.E-02	1.30.E+00	8.10.E-02	1.41.E+00	100
	Polished rice	(unknown)	-	2016/5/25	5.56.E-01	3.32.E-02	1.07.E-01	1.75.E-02	4.49.E-01	2.82.E-02	5.47.E-01	100
	Polished rice	from Naraha, Fukushima	16	2015/11/20	9.14.E-01	1.82.E-02	1.71.E-01	7.51.E-03	7.44.E-01	1.66.E-02	7.43.E-01	99.9
	Brown rice	from Shiroi, Chiba	201	2016/8/31	1.65.E+00	1.80.E-02	2.49.E-01	1.74.E-02	1.40.E+00	4.49.E-03	1.38.E+00	98.8
	Rice bran	from Shiroi, Chiba	201	2016/6/4	1.09.E+01	1.78.E-01	1.75.E+00	6.35.E-02	9.20.E+00	1.67.E-01	9.01.E+00	98.0
	Polished rice	from Gumma	230	2016/3/17	8.90.E-02	4.08.E-03	1.44.E-02	2.12.E-03	7.46.E-02	3.49.E-03	6.95.E-02	93.2
	Polished rice	from Namie, Fukushima	11	2015/12/4	8.54.E-01	3.66.E-02	1.46.E-01	1.54.E-02	7.08.E-01	3.32.E-02	6.45.E-01	91.1
	Brown rice	from Akiha, Niigata	174	2015/4/22	8.14.E-02	1.11.E-02	3.54.E-02		8.14.E-02	1.11.E-02		-
	Polished rice	(unknown)	-	2015/3/9	1.25.E-01	1.37.E-02	3.28.E-01		1.25.E-01	1.37.E-02		-
Seasonings	Soup stock	by Riken company	-	2015/12/22	1.32.E-01	2.09.E-02	8.15.E-02		1.32.E-01	2.09.E-02		-
	Soup stock	by Riken company	-	2016/3/15	1.37.E-01	2.49.E-02	7.94.E-02		1.37.E-01	2.49.E-02		-
	Curry powder	(unknown)	-	2016/5/23	5.29.E-01	4.63.E-02	1.61.E-01		5.29.E-01	4.63.E-02		-
Seaweeds	*Wakame* seaweed	from Iwate	254	2016/5/20	1.35.E-01	2.45.E-02	1.02.E-01		1.35.E-01	2.45.E-02		-
	Dried green lavers	from Kochi	870	2016/2/15	1.80.E-01	4.32.E-02	1.53.E-01		1.80.E-01	4.32.E-02		-
	Dried green lavers	from Aichi, Mikawa Bay	452	2016/1/25	4.51.E-01	7.40.E-02	2.56.E-01		4.51.E-01	7.40.E-02		-
water	Tap water	from Nerima, Tokyo	225	2016/6/2	1.86.E-03	3.93.E-04	1.86.E-03		1.86.E-03	3.93.E-04		-
Bean & Bean product	*Miso* (Soybean paste)	by marukome	-	2014/7/26	5.32.E-02	5.52.E-03	1.34.E-02	2.91.E-03	3.99.E-02	4.69.E-03	3.85.E-02	96.6
	Soy Sauce	by Kikkoman Corporation	-	2014/7/11	5.20.E-02	6.90.E-03	2.30.E-02		5.20.E-02	6.90.E-03		-
	Azuki beans	from Hokkaido	628	2014/9/22	1.07.E-01	1.12.E-02	4.02.E-02		1.07.E-01	1.12.E-02		-
Beverages	Green tea	from Shizuoka	325	2014/5/30	1.40.E-01	1.33.E-02	4.59.E-02	9.23.E-03	9.36.E-02	9.54.E-03	1.26.E-01	100
	Beer	by Suntory Holdings Limited	-	2014/9/5	8.37.E-03	2.11.E-03	5.57.E-03		8.37.E-03	2.11.E-03		-
	Juice (orange)	from Ehime	850	2014/10/7	1.44.E-02	3.63.E-03	9.66.E-03		1.44.E-02	3.63.E-03		-
	Juice (apple)	from Aomori	415	2014/9/1	3.29.E-02	5.77.E-03	2.06.E-02		3.29.E-02	5.77.E-03		-
Cereals	Cereal	by Kellogg(Japan)G.K.	-	2014/10/3	9.33.E-02	7.93.E-03	2.77.E-02	4.32.E-03	6.56.E-02	6.65.E-03	8.47.E-02	100
Fish	Japanese pond smelt	from Ibaraki	167	2014/11/27	3.19.E+01	7.90.E-01	7.16.E+00	3.21.E-01	2.47.E+01	7.21.E-01	2.31.E+01	93.3
	Salmon	from Hokkaido	490	2014/11/14	1.19.E-01	6.12.E-03	1.61.E-02	4.12.E-03	1.03.E-01	4.53.E-03	5.09.E-02	49.2

	Fish	Cod	from Hokkaido	629	2014/12/26	6.46.E-03	9.99.E-04	4.23.E-03		6.46.E-03	9.99.E-04		-
		Niboshi (Dried sardines)	from Nagasaki	1141	2014/11/21	1.55.E-01	1.73.E-02	6.78.E-02		1.55.E-01	1.73.E-02		-
	Fruits	Persimmon	from Tokyo	222	2014/12/10	6.48.E-01	5.45.E-02	1.59.E-01	3.31.E-02	4.90.E-01	4.33.E-02	5.13.E-01	100
		Apple (fruit)	from Gumma	190	2014/10/8	1.21.E+00	1.05.E-01	3.16.E-01	6.62.E-02	8.96.E-01	8.14.E-02	9.69.E-01	100
		Apple (peel)	from Gumma	190	2014/10/8	5.38.E-01	6.57.E-02	1.42.E-01	3.93.E-02	3.96.E-01	5.27.E-02	4.35.E-01	100
		Apple (core)	from Gumma	190	2014/10/8	1.45.E+00	5.83.E-02	3.31.E-01	2.96.E-02	1.12.E+00	5.02.E-02	1.02.E+00	91.1
		Blueberries	from Ibaraki	190	2014/9/23	8.14.E-01	7.42.E-02	2.03.E-01	3.85.E-02	6.10.E-01	6.34.E-02	6.17.E-01	100
	Green vegetables	Chinese cabbage	from Iwate	254	2014/12/6	1.20.E-02	3.43.E-03	9.51.E-03		1.20.E-02	3.43.E-03		-
	Meat	Deer Meat	from Yamanashi	300.2	2015/1/26	6.69.E+00	2.35.E-01	1.10.E+00	9.86.E-02	5.59.E+00	2.13.E-01	3.72.E+00	66.5
		Chicken Meat	from Iwate	250	2014/6/30	2.29.E-02	6.77.E-03	1.97.E-02		2.29.E-02	6.77.E-03		-
	Milk & Milk products	Milk	from Ibaraki	185	2014/7/18	1.14.E+00	1.44.E-02	2.92.E-01	6.34.E-03	8.45.E-01	1.29.E-02	8.36.E-01	99.0
		Milk	from Aomori	356	2015/2/17	1.38.E-01	6.79.E-03	2.16.E-02	2.82.E-03	1.17.E-01	6.18.E-03	7.41.E-02	63.4
2014		Yoghurt	by MEGMILK SNOW BRAND Co.,Ltd.	-	2014/8/25	1.88.E-01	6.97.E-03	2.11.E-02	2.75.E-03	1.67.E-01	6.40.E-03	6.22.E-02	37.2
		Milk	from Hokkaido	629	2015/1/10	2.12.E-02	2.94.E-03	1.07.E-02		2.12.E-02	2.94.E-03		-
		Milk	by Morinaga company	-	2014/8/9	2.89.E-02	4.12.E-03	1.51.E-02		2.89.E-02	4.12.E-03		-
		Milk	from Hokkaido	629	2015/1/30	2.91.E-02	2.14.E-03	7.35.E-03		2.91.E-02	2.14.E-03		-
	Mushrooms	Eryngii mushroom	from Yamanashi	293	2015/1/17	7.48.E-01	7.35.E-02	1.96.E-01	5.55.E-02	5.52.E-01	4.82.E-02	6.56.E-01	100
		Maitake mushroom	from Tokyo	253	2014/11/29	7.54.E+00	2.94.E-01	1.83.E+00	1.22.E-01	5.72.E+00	2.68.E-01	5.87.E+00	100
		Maitake mushroom	from Gumma	215	2014/10/15	1.85.E+02	2.83.E+00	4.48.E+01	1.18.E+00	1.40.E+02	2.57.E+00	1.38.E+02	98.5
		Maitake mushroom	from Gumma	250	2015/1/13	1.52.E+02	2.23.E+00	3.35.E+01	8.50.E-01	1.19.E+02	2.06.E+00	1.12.E+02	93.9
		Maitake mushroom	from Yamanashi	293	2015/1/14	2.31.E+01	1.10.E+00	3.48.E+00	4.55.E-01	1.96.E+01	9.98.E-01	1.16.E+01	59.2
	Nuts and Seeds	Almond	from Spain	10660	2014/12/20	6.27.E-02	1.39.E-02	5.37.E-02		6.27.E-02	1.39.E-02		-
		Coffee	(unknown)	-	2015/2/27	2.92.E-01	3.72.E-02	1.12.E-01		2.92.E-01	3.72.E-02		-
		Cocoa	by KATAOKA & Co., Ltd. /VAN HOUTEN COCOA	-	2015/1/5	3.26.E-01	5.39.E-02	1.42.E-01		3.26.E-01	5.39.E-02		-
		Coffee	mixed from Indonesia and Brazil	-	2015/29	4.25.E-01	8.18.E-02	2.80.E-01		4.25.E-01	8.18.E-02		-
	Other vegetables	Lotus Root	from Ibaraki	164	2015/1/5	2.19.E-01	1.37.E-02	5.36.E-02	6.32.E-03	1.65.E-01	1.21.E-02	1.78.E-01	100
		Kumazasa (Bamboo Leaves)	from Yamanashi	276	2015/1/20	2.54.E+01	9.55.E-01	6.17.E+00	4.14.E-01	1.93.E+01	8.60.E-01	2.07.E+01	100
		Lotus Root	from Ibaraki	167	2014/12/3	1.45.E+01	2.22.E-01	3.42.E+00	8.41.E-02	1.10.E+01	2.05.E-01	1.10.E+01	99.6
		Green Tea Leaf	from Izu, Shizuoka	325	2014/5/30	2.13.E+00	4.50.E-01	1.30.E+00		2.13.E+00	4.50.E-01		-
	Rice	Polished rice	from Tochigi	140	2014/8/26	2.22.E-01	1.54.E-02	5.65.E-02	7.95.E-03	1.65.E-01	1.32.E-02	1.67.E-01	100
		Rice Bran	from Fukushima	80	2014/12/22	4.33.E+00	1.10.E-01	1.04.E+00	4.71.E-02	3.29.E+00	9.89.E-02	3.39.E+00	100
		Polished rice	from Fukushima	7.5	2015/1/7	1.66.E+00	5.24.E-02	3.82.E-01	2.09.E-02	1.28.E+00	4.80.E-02	1.27.E+00	99.2
		Polished rice	from Fukushima	7.5	2015/1/8	2.40.E+00	6.02.E-02	5.26.E-01	2.29.E-02	1.87.E+00	5.57.E-02	1.75.E+00	93.4
	Processed	Curry powder	by HOUSE FOODS CORPORATION	-	2014/8/11	4.13.E-02	6.08.E-03	2.53.E-02		4.13.E-02	6.08.E-03		-
		Soup stock	(unknown)	-	2015/2/22	1.61.E-01	2.22.E-02	8.04.E-02		1.61.E-01	2.22.E-02		-
